# A combined electro-optical deformability micro-cytometer†

**DOI:** 10.1039/d4ra04800h

**Published:** 2024-10-29

**Authors:** Xueping Zou, Daniel C. Spencer, Junyu Chen, Hywel Morgan

**Affiliations:** a School of Electronics and Computer Science Southampton SO17 1BJ UK; b Institute for Life Sciences, University of Southampton Southampton SO17 1BJ UK hm@ecs.soton.ac.uk

## Abstract

We have developed a deformability cytometer that simultaneously measures the optical and electrical shape change of single cells in a viscoelastic shear flow. The optical deformability of single cells is measured using a low-cost CMOS camera illuminated with a high-power LED triggered from an electrical impedance signal created by a passing cell. Simultaneously the electrical deformability of the cell is determined using electrode arrays that measure shape changes along different axes. This is achieved by correlating the optical and electrical signals captured without complicated synchronisation. The system was characterised by measuring the deformability of HL-60 cells treated with cytochalasin D, latrunculin B and glutaraldehyde. Results demonstrate excellent correlation between the optical and electrical methods.

## Abstract 2

This paper describes a cytometer that measures the optical and electrical deformabilitiy of multiple single cells in a viscoelastic flow.

## Graphical abstract

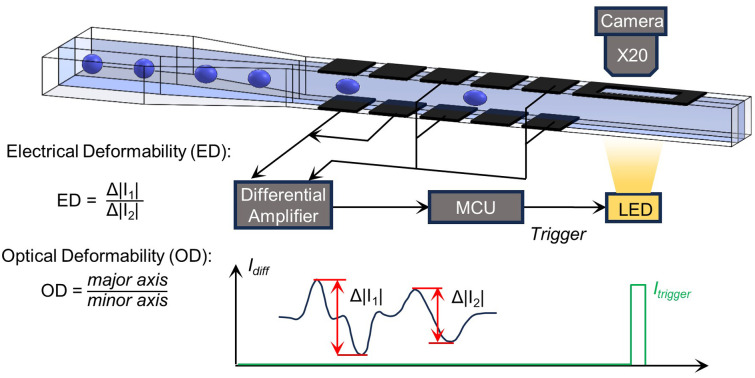

## 1. Introduction

Many studies have demonstrated the wide degree of heterogeneity in cellular systems and shown that inherent biophysical markers can be used to distinguish different cell types, state and function. Bio-mechanical and bio-electrical properties are biophysical markers that have been linked to disease presentation, progression and treatment. Cells can exhibit distinct phenotypes depending on external interventions or the micro-environment, and measuring the heterogeneity of cells based on biophysical properties such as size, shape, mechanical and electrical characteristics provides a rapid label-free characterisation method. The deformability of cells is of particular interest and cell mechanical properties can be used for label-free differentiation.^1–3^ For example, as cells become malignant the cytoskeleton transforms from a rigid structure to a deformable state^4–7^ and the stiffness of invasive cancer cells is related to metastatic potential.^8,9^ Conventional methods of measuring cell stiffness such as atomic force microscopy,^10^ micropipette aspiration,^11^ optical stretching^12^ and magnetic bead rheology^13^ are technically demanding and low-throughput.^14–16^ To address these issues microfluidic deformability cytometry has been developed, where cell shape is measured using high speed optical systems to determine deformability.^17^

Shear deformability cytometry (sDC) uses a viscoelastic shear flow to create a hydrodynamic force on cells,^16,18,19^ causing deformation without the cell contacting the channel. Constriction channels have also been used to measure deformability where cells squeeze through a narrow channel and their transit time is measured optically or electrically.^20–26^ However, this method requires a narrow microchannel (smaller than the cell), and the transit time can be influenced by cell–wall interaction, and the device is prone to blockage.

Impedance methods have recently been developed to measure cell mechanical properties using shear flow deformability.^27,28*a*^ In this work, we describe a deformability cytometer that measures the shape change of a cell using both an optical and electrical technique. Cells are suspended in a viscoelastic fluid (0.5% (w/v) methylcellulose) and flow along a narrow channel where a shear stress deforms them. The electrical impedance of the cell is determined using arrays of micro-electrodes that measure cell impedance along two orthogonal axes.^28*b*^ As the cells deform due to viscous stress, the shape change is determined from the ratio of these impedance signals. Simultaneously the optical deformability of each cell was determined using the impedance signal to trigger a high-speed LED that projects an image of each cell onto a CMOS camera. To validate the system, experiments were performed using HL60 cells treated with drugs that perturb the cytoskeleton (cytochalasin D and latrunculin B), reducing the stiffness of the cells, and also with glutaraldehyde which stiffens the cells. The viscoelastic fluid partly-focuses cells^29,30^ minimising the positional influence on the impedance signals.^31*a*^ However, in the absence of a sheath flow not all cells flow through the centre and optical images of off-centre cells show an asymmetric deformation which can skew the data. This artefact is not observed in the electrical impedance data. Excellent correlation between the electrical and optical deformability data was demonstrated.

### 1.1 System overview

**Fig. 1:**
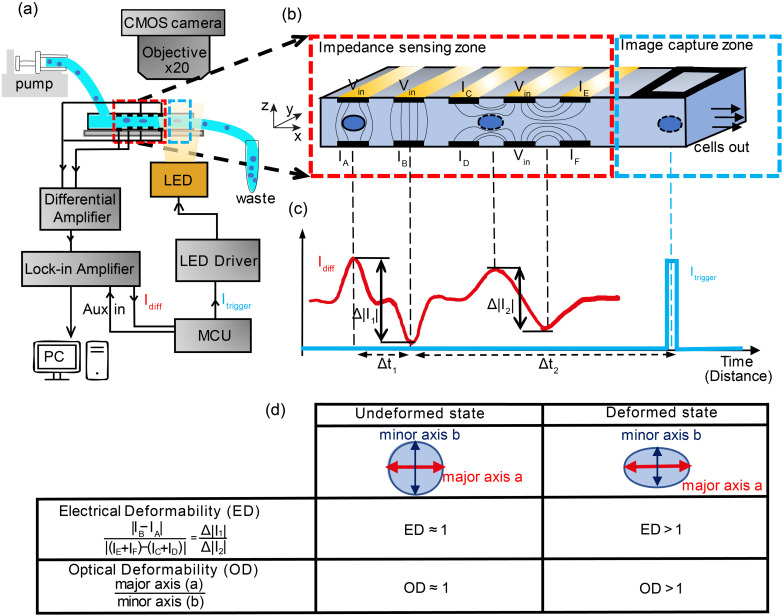
System overview. (a) Cells suspended in a viscoelastic fluid are pumped through a microfluidic channel (40 μm × 30 μm) where they deform and become elongated. The chip contains an array of micro-electrodes that measures the shape of the cell along two different axes. The electrodes are connected to amplifiers and a lock-in to extract the impedance signals. The lock-in also provides a trigger signal. (b) The channel is divided into two parts: the electrical impedance sensing zone and the optical image capture zone. The two electrode configurations measure the vertical (Δ|*I*_1_|) and horizontal (Δ|*I*_2_|) dimensions of the cell. An image is focused onto a simple CMOS camera with a ×20 objective. (c) The trigger from the lock-in sends a pulse to a high intensity LED creating an image in the camera. (d) Definition of Electrical and Optical Deformability (ED & OD).

Fig. 1(a) shows the experimental setup. Cells suspended in a viscoelastic fluid are pumped along a microfluidic channel (40 μm × 30 μm cross-section) passing between an array of electrodes generating impedance signals which are used to characterise the electrical deformability. Synchronised optical images of deformed cells are captured by triggering a high-speed LED which projects an image onto a low-cost CMOS camera (MQ003CG-CM, XIMEA). Impedance based triggering of a camera has been previously demonstrated.^31*b*^where an electrical signal from a cell crossing a set of electrodes was used to trigger a camera that captured 15 successive frames with a high-speed camera and a 50 μs exposure time. Here we adopt a different approach that includes a variable time delay to trigger an LED so that one cell is captured on a single frame. The impedance signal is first demodulated with a lock-in amplifier (HF2LI, Zurich Instruments), before the digitised signal is sent to a microcontroller (Teensy 4.0, PJRC). The microcontroller determines the velocity of the cell and calculates the time-point at which it arrives at the image capture window (Δ*t*_2_), as shown in the figure. At this point an LED (CBT-90-B-L11, Luminus) is triggered by the microcontroller illuminating the image capture zone, projecting the cell outline onto the camera set to video recording mode (100 fps). Although the camera has a low frame rate, motion blur is eliminated by using a short (2 μs) illumination pulse generated using a MOSFET driver to provide the required high-current to the LED.^32*a*,*b*^ Thus each frame contains an image of a cell, synchronised to the electrical impedance signal.

The key to synchronous measurement of the electrical and optical data is the triggering. As illustrated in Fig. 1(b), the impedance cytometer uses two different electrode configurations. The first configuration consists of two pairs of opposing electrodes, whilst the second consist of two pairs of coplanar electrodes. Both sets operate in differential mode to eliminate drift and noise in the signal.^33^ The vertical arrangement (*I*_A_ and *I*_B_) measures cell volume whilst the horizontal set measures cell deformation in the direction of the flow. The differential current waveform generated by the electrode pairs consists of two consecutive anti-symmetric double Gaussians (Fig. 1(c)), the first pair generated by electrode configuration 1 and the second by configuration 2. The microcontroller uses the signal from configuration 1 to calculate the transit time and average velocity of each cell and to trigger the LED ensuring that the electrical and optical deformability of each cell are correlated.

Fig. 1(d) summarises how the electrical deformability (ED) and optical deformability (OD) are calculated. At low frequencies cells are electrically insulating so that the impedance (magnitude of current Δ*I*_1_ and Δ*I*_2_) is a function of particle cross section when observed from two different projections. In other words, these signals measure the shape of the object along the minor and major axes. The electrical deformability is, therefore, the ratio of the impedance (current) from electrode configuration 1 and configuration 2 (ref. 28*b*), *i.e.*
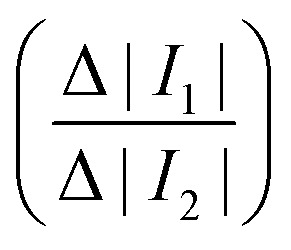
. Optical images of cells obtained from the camera were post-processed in MATLAB using consecutive functions to estimate the outline of a cell and identify the major axis and minor axis. Raw images were first contrast enhanced and converted into grayscale images, with edge detection applied to determine the cell perimeter. The object outline was then fitted to an ellipse by computing second-order moments of this region using the MATLAB function *regionprop*. This gives the major axis, minor axis and centroid position. The Optical Deformability (OD) of a cell is the ratio of major axis to minor axis. For solid objects, the deformability is 1 whereas deformed cells have deformability larger than 1.

### 1.2 Trigger mechanism

**Fig. 2:**
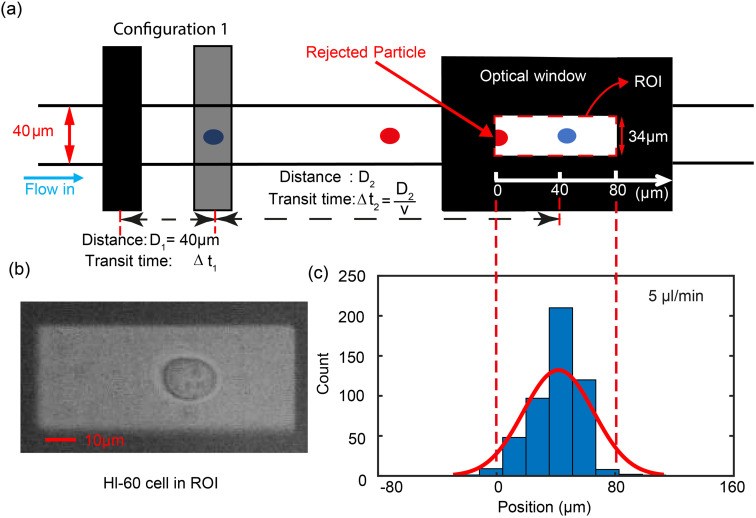
Diagram showing the principle of the triggering mechanism. (a) The velocity of a cell is obtained from ratio of the distance between impedance peaks (*D*_1_) and the transit time (Δ*t*_1_) obtained from the impedance signal. The predicted time to trigger Δ*t*_2_ is then calculated and the trigger pulse generated to drive the LED. The red rectangle defines the ROI (34 μm wide) of the optical image capture zone. Only the first configuration electrodes are shown in the diagram (the other set is not used for triggering). (b) Image of a deformed HL-60 cell in the ROI. (c) Histogram of experimental cell position when illuminated by the LED. Data for 500 cells at flow rate of 5 μl min^−1^.

The triggering principle is shown in further detail in Fig. 2(a). Rather than using a simple threshold trigger, the time at which a cell arrives at the imaging zone is calculated from its velocity (transit time). The microcontroller samples the impedance signal from electrode configuration 1 *via* an ADC at a sample rate of 87 ksps. This data stream is processed by a peak-valley detection algorithm to determine the transit time Δ*t*_1_. The peak and valley of the impedance signal corresponds to the centre of the two pairs of electrodes (*D*_1_ = 40 μm) allowing the velocity to be calculated 
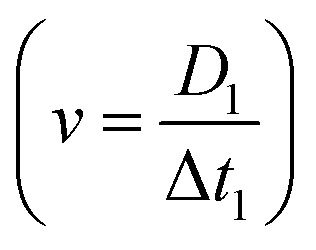
. The distance to the centre of the optical window *D*_2_ is known, so that the transit time Δ*t*_2_ can be calculated. Ideally, the microcontroller should illuminate the particle in the centre of the zone (Fig. 2(b)), but a small random offset in position occurs due to latency in the microcontroller. Fig. 2(c) shows the experimental distribution of cell position in the optical window. The peak is close to the centre of the window (+40 μm) so that the vast majority of the cells are captured by the camera.

Coincidence in the impedance data is very low. For example, at a cell concentration of 500 000 cells per ml, the Poisson distribution *P*(*k*) = *λ*^*k*^e^−*λ*^/*k*! shows that the probability of two coincident particles is 2%, therefore the cell concentration was set to 100 000 cells per ml where the probability of coincidence is nearly zero. Practically, the coincidence rate is dominated by the frame rate of the camera (100 fps). To determine the optical coincidence rate, a set of images was analysed at a flow rate of 5 μl min^−1^. This gave a coincidence rate of around 2% (1.9% for 53 frames with two cell from a total of 2844). Increasing the flow rate would lead to a higher coincidence rate so that values between 5, 10 and 15 μl min^−1^ were used. Increasing the flow rate will also lead to blurring of the image. At 5 μl min^−1^, the maximum velocity of a cell is 0.144 m s^−1^ meaning it moves 0.288 μm in 2 μs (LED pulse length). At 15 μl min^−1^ this increases to 0.864 μm. For a 12 μm diameter cell, the maximum blur is about 7% of the diameter. Experimentally no blurring is seen at 5 μl min^−1^, whilst at 15 μl min^−1^ some image blurring is apparent. However the image processing accommodates a small degree of blurring and accurately detects the edge of cells up to 15 μl min^−1^, but not at higher flow rates.

### 1.3 Correlation of optical and electrical measurement

**Fig. 3:**
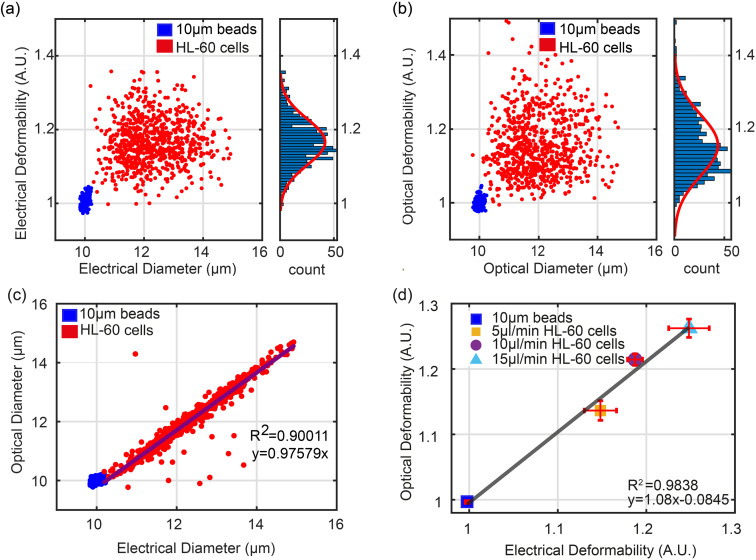
Correlation between electrical and optical properties of HL60 cells and beads. (a) and (b) are scatter plots of Electrical and Optical Diameter *versus* Optical and Electrical Deformability, together with histograms of deformability. 10 μm polystyrene beads were used as calibration particles for both size and deformability. HL60 cells were untreated, and the flow rate was 5 μl min^−1^. (c) Electrical *vs.* Optical Diameter can be fitted to a linear equation *y* = 0.976*x*, with *R*^2^ = 0.90. (d) Correlation between the mean value of ED and OD for beads and HL-60 cells at 5 μl min^−1^, 10 μl min^−1^ and 15 μl min^−1^ flow rates (data is the mean of three repeats *n* = 3).

In order to characterise the system, the deformability of HL60 cells was measured. First a sample of untreated HL60 cells was suspended in Methylcellulose buffer (0.5% w/v in DPBS) together with 10 μm diameter solid polystyrene calibration beads (of known electrical properties). Fig. 3(a) and b show scatter plots of ED and OD *vs.* electrical and optical diameter for beads and cells at a flow rate of 5 μl min^−1^. The electrical diameter was calculated from the cube root of the impedance measured from the first electrode configuration, referenced to the calibration particles. This electrode array accurately measures particle volume and is less influenced by shape.^28*a*^ Optical diameter was calculated as described above 
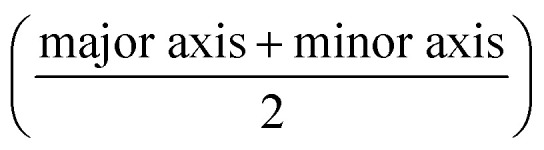
, again referenced to the calibration beads. The two plots show that as anticipated the cells deform due to the shear flow but that the beads do not and have a deformability of 1.0.

Fig. 3(c) shows a plot of the correlation between the electrical diameter and optical diameter after system calibration using solid particles of known size. The impedance signals for the cells measured by the two different electrode configurations were normalised against the 10 μm polystyrene beads.^34^ These provide reference values for both deformability and electrical impedance, and eliminate any drift in the system. The optical system was calibrated using the 10 μm beads so that the pixel number could be converted into absolute dimensions. As shown in Fig. 3(c) there is excellent correlation between optical and electrical measurements for the wider range of cell diameters. This data is for a flow rate of 5 μl min; results for 10 μl min^−1^ and 15 μl min^−1^ are shown in Fig. S1.†

Fig. 3(d) shows the correlation (*R*^2^ > 0.9) between the mean value of OD and ED for beads and untreated cells at different flow rates (5 μl min^−1^, 10 μl min^−1^, 15 μl min^−1^) from three repeat experiments. As flow rate increases, cells are exposed to a higher shear stress leading to a higher deformability. The trend is linear (*y* = 1.08*x* − 0.0845) again with excellent correlation between the two techniques.

Fitting the data in Fig. 3a and b to a normal distribution shows that the optical deformability has a higher variance than electrical (*N*_ED_ ∼ (1.167 ± 0.06), *N*_OD_ ∼ (1.156 ± 0.08)). To explore the reason for this, HL60 cells were treated with cytochalasin D and with latrunculin B. Cytochalasin D (Cyto-D) attaches to the barbed ends of actin filaments, preventing actin filament elongation, limiting polymerization and causing actin filaments to break down, resulting in cytoskeletal structure loss. Latrunculin B (Lat-B), unlike cytochalasin, binds actin monomers in a 1 : 1 stoichiometry, blocking polymerization with actin filaments.

**Fig. 4:**
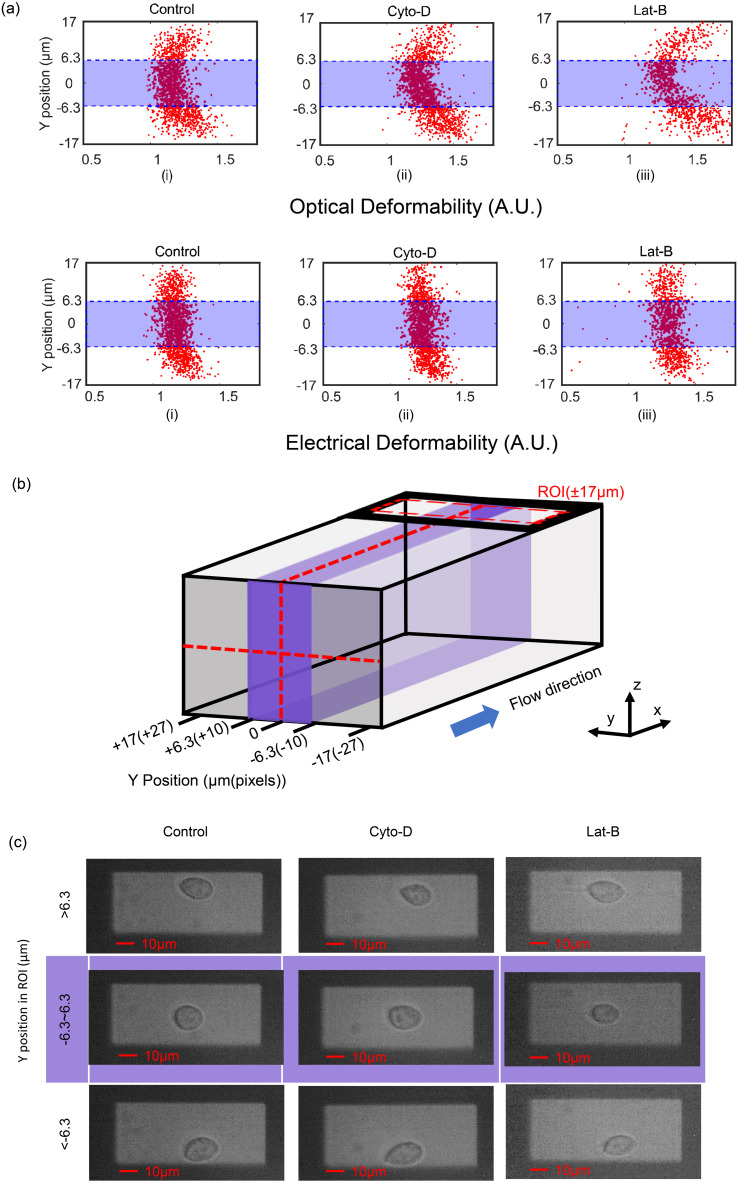
(a) Comparison between OD and ED for HL60 cells treated with Cyto-D and Lat-B demonstrating how cell deformation depends on lateral position in the channel. Top row shows optical deformation scatter data for untreated and treated cells. The scatter plot has a parabolic shape. Bottom row shows the scatter plot of electrical deformability. The shaded box (−6.3 μm to +6.3 μm) defines the ROI within which cells were measured. Those near the walls were discarded. Flow rate was 10 μl min^−1^. (b) Schematic diagram showing channel cross section and dimensions. (c) Images of cells located at different Y-position in the ROI (−17 μm to +17 μm) and different treatment. Cells were treated with 1 μM Cyto-D and 25 nM Lat-B.

The optical and electrical deformability data is summarised in Fig. 4(a), where the deformability is shown to vary with Y-position across the channel width. Consistent with previous reports^17,35^ treated cells have a higher deformability than untreated cells. However, the optical deformability in particular is influenced by the position of the cell across the channel width. It is known that the viscoelastic fluid focuses particles into the central region (along the *y*-axis)^28*b*,29,30^ minimising the variation in the impedance signals along the *z*-axis. However, the data in Fig. 4(a) clearly shows that many cells also flow close to the channel wall (along the *y*-axis, see Fig. 4(b)). Representative images of the three different populations of cells at three different positions in the optical ROI (refer to Fig. 4(b)) are show in Fig. 4(c). Images (top row Fig. 4(c)) shows that cells near the channel wall deform into tear-drop shapes, presumably due to unbalanced shear forces near the wall. 2D image processing of these cells includes the shape of the tail and tends to skew the long axis measurement of the cell, and therefore the mean OD. These off-centre cells have a higher OD compared with cells flowing close to the midline. This distortion in the cell shape is reflected in the OD scatter plots of Fig. 4(a), which has a parabolic shape because cells closest to the wall deform the most and are found at the extremes of the parabola.

The optical deformability data can be contrasted with the electrical deformability which appears to be independent of the Y-position. The asymmetric tear-drop shape has a minor influence on the electrical impedance so that the measured ED is far less dependent on the Y-position of the cells in the channel (Fig. 4(a) bottom row). Post-processing the data to eliminate the slower moving cells near the channel walls demonstrates excellent correlation between the ED and OD. A boundary of ±10 pixels either side of the channel centre line (approximately ±6.3 μm) was defined and only cells within this region (purple region in Fig. 4(b)) were analysed.

**Fig. 5:**
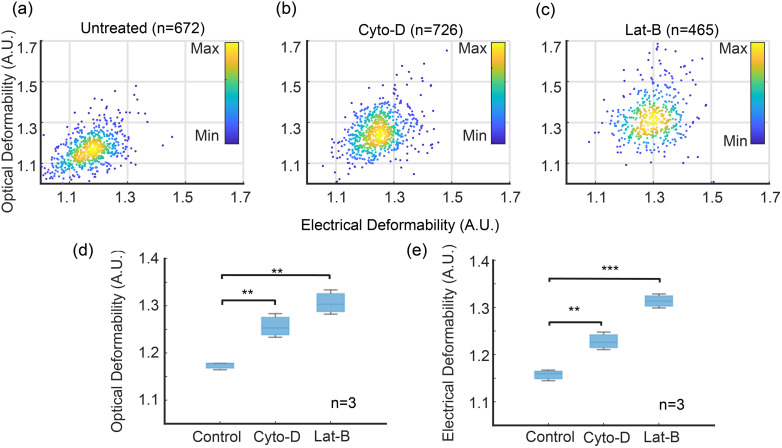
Optical and electrical deformability of HL60 cells treated with Cyto-D and Lat-B. The optical results are filtered by Y-position in the channel to exclude cells that are close to the channel walls, *i.e.* outside the shaded region in Fig. 4. (a)–(c) are scatter plots for 672 out of 1430 (47%) untreated cells, 726 out of 1511 (48%) Cyto-D treated cells, 465 out of 1095 (42%) Lat-B treated cells. (d) and (e) are box charts of electrical and optical deformability (*n* = 3). Data are mean ± SD. **p ≤ 0.01, ***p ≤ 0.001 (*t*-test), compared with the untreated group. Flow rate 10 μl min^−1^. ED of untreated = 1.16 ± 0.011; Cyto-D: 1.23 ± 0.019; Lat-B: 1.31 ± 0.015 and OD (untreated): 1.18 ± 0.008, Cyto-D: 1.25 ± 0.025, Lat-B: 1.30 ± 0.026.

Fig. 5(a)–(c) shows scatter plots of ED *vs.* OD for control and treated cells. As expected, untreated cells have the lowest deformability whilst the Lat-B treated cells have the largest deformability. The correlation between the two techniques is excellent. Fig. 5(d) and (e) show OD and ED data (mean ± SD) for HL60 cells treated with Cyto-D and Lat-B, compared with untreated cells showing that deformability determined by impedance is equivalent to the optical method. For example at a flow rate of 10 μl min^−1^, the ED of untreated cells = 1.16 ± 0.011, compared with OD = 1.18 ± 0.008.

### 1.4 Cross-linking with glutaraldehyde

**Fig. 6:**
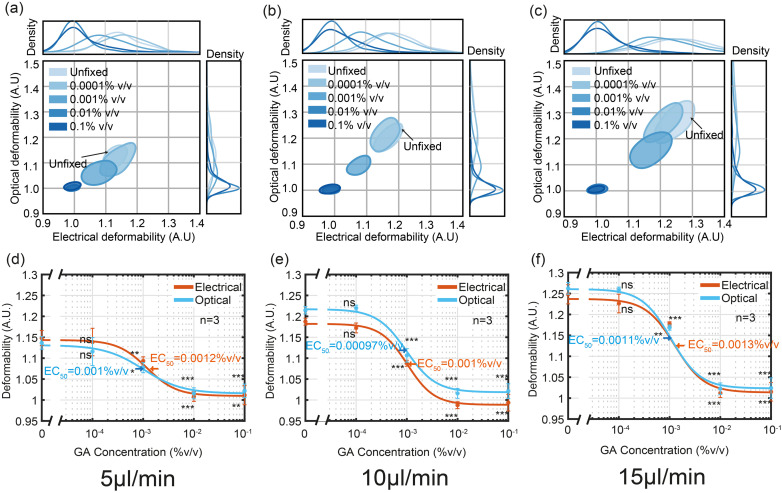
Contour plots and dose–response curves of ED *vs.* OD for HL60 cells treated with different concentrations of glutaraldehyde. The data was collected at three different flow rates 5 μl min^−1^, 10 μl min^−1^ and 15 μl min^−1^ (a)–(c) are 50% density contours plots of ED *vs.* OD at 5, 10, and 15 μl min^−1^ respectively. (d)–(f) are dose–response curves (*p ≤ 0.05, **p ≤ 0.01, ***p ≤ 0.001, *t*-test). Data is the mean of three biological repeats, *n* = 3.

As a final set of experiments the performance of the electro-optical impedance cytometer was evaluated using HL60 cells treated with different concentrations of glutaraldehyde (GA) which cross-links the cell proteins making them stiffer. Fully crosslinked cells have a deformability similar to solid beads, close to 1. Fig. 6 shows OD *vs.* ED as a function of GA concentration plotted as 50% contour plots for three different flowrates (a) 5 μl min^−1^ (a) 10 μl min^−1^ and (a) 15 μl min^−1^. Fig. 6(a)–(c) shows that at the lowest concentration 0.0001% (v/v), both the ED and OD of cells are similar to those of untreated cells, and that the degree of deformation increases with flow rate. For each flow rate the half-maximal concentration (EC_50_) was determined by fitting the data to the three-parameter Hill equation. The values for the two different techniques were almost identical (OD: 0.001%, 0.00097% and 0.0011% (v/v) ED: 0.0012%, 0.001% and 0.0013% (v/v)) and are similar to those reported elsewhere.^35^

## 2. Conclusion

We have developed a simple shear flow electro-optical deformability cytometer. The system was used to measure the deformation of HL60 cells treated with Cyto-D, Lat-B and GA. The device uses a simple inexpensive CMOS camera to capture image of cells at a throughput of a few tens of cells per second. As a cell moves down a channel the electrical impedance signal is used to trigger a high intensity LED which projects an image of a deformed cell onto the camera. The electrical deformability is determined from the ratio of the electrical impedance measured along two different orthogonal axes. Experiments show a very high correlation between both methods. The system does not use a hydrodynamic sheath flow but relies on the viscoelastic fluid to partially focus cells into a plane in the centre of the channel. Although this simplifies the system, it means that some particles close to the channel wall are asymmetrically distorted leading to errors in the optical images. This leads to a positional dependence in the scatter data which can be corrected by post-processing, excluding all cells that flow close to the channel walls. Interestingly the electrical deformability appears to be much less affected by the position of the cells in the channel. The technique is simple and the correlation between ED and OD is extremely high.

## 3. Experimental methods

### 3.1 Device fabrication

The chip fabrication is described in detail elsewhere.^36^ Platinum electrodes were lithographically patterned onto glass wafers and channels made from Perminex. Two wafers were thermo-compression bonded and scribed into individual chips. The holder was made from PEEK, and provides fluid and electrical connections.

### 3.2 Measurement procedure

Prior to measurement, all buffers were filtered through a 0.22 μm filter to avoid blockage of the chip. The chips were cleaned before experiment with 1 M sodium hydroxide solution at a low flow rate (typically 5 μl min^−1^) for about 10 minutes. After cleaning, the channel was flushed with deionised water (DI water). For each experimental condition, measurements were recorded after the flow had stabilized for 2 minutes.

### 3.3 Cell culture

HL60 cells were grown in RPMI 1640 + Glutamax (Gibco) media with 10% Fetal Bovine Serum (FBS, Gibco), 1% Penicillin-Streptomycin (Sigma-Aldrich) in a humidified incubator at 37 °C and 5% CO_2_. To minimize the influence of variation between cell batches, different experimental groups shared a common stock of cell sublines and measurements were performed within ten passages. To further standardize growth conditions, the same serum batches of cells were used, following the same subculture protocol, and were harvested for measurements in the concentration range (0.5–1 × 10^6^ cells per ml).

### 3.4 Methylcellulose (MC) solution preparation

0.5% (w/v) MC-DPBS buffer was used for suspending cells. 200 ml of 0.5% (w/v) MC-DPBS solution was made as follows. After heating 70 ml of DPBS in a clean beaker to 80 °C, 1 g of MC powder was added and stirred gently to disperse. 130 ml DPBS at room temperature was added to the mixture with constant stirring to avoid clumping or aggregation. The MC mixture hydrates as the temperature decreases and becomes a jelly. After the solution has cooled to room temperature, the mixture was stored at 4 °C to fully hydrate the MC and avoid bacterial growth. The final suspension was filtered before use.

### 3.5 Cytoskeletal disruption

HL60 cells were treated with Cyto-D (Sigma Aldrich) and Lat-B (Sigma Aldrich). HL60 cells in 1 ml volume at a concentration of about 5 × 10^5^ cells per ml were centrifuged and resuspended in 0.5% w/v MC solution. Cyto-D and Lat-B were added to the cell suspension to a concentration of 1 μM and 25 nM respectively. Stock solutions of Cyto-D and Lat-B were made in DMSO. This stock was diluted to ensure the same concentration of DMSO in each group of cells, which is 0.5% (v/v). Cyto-D and its untreated control group (DMSO exposed) were kept at 37 °C in an incubator for 10 minutes; Lat-B and its untreated control group were kept for 30 minutes. After incubation, the samples were loaded into the syringe for measurement.

### 3.6 Glutaraldehyde cross linking

To fix cells with glutaraldehyde (GA), cells were harvested at 5 × 10^5^ cells per ml and suspended in DPBS. Five different groups of cells were processed in pure DPBS with different concentrations of GA-DPBS solutions (0.0001%, 0.001%, 0.01% and 0.1% v/v), and kept at room temperature for 30 minutes. After incubation, cells were centrifuged and resuspended in 0.5% w/v MC solution prior to deformability measurements.

## Data availability

Data for this article are available at https://doi.org/10.5258/SOTON/D3152.

## Conflicts of interest

No conflicts of interest.

## Supplementary Material

RA-014-D4RA04800H-s001

RA-014-D4RA04800H-s002
