# Supplementary material for: A combined electro-optical deformability micro-cytometer
Source: RSC Adv. 2024 Oct 29;14(46):34270–8. doi: 10.1039/d4ra04800h (PMC11520318; doi:10.1039/d4ra04800h)
Supplement: RA-014-D4RA04800H-s002 [file RA-014-D4RA04800H-s002.pdf]

Supplementary Information (SI) for RSC Advances.
This journal is © The Royal Society of Chemistry 2024

Supplementary

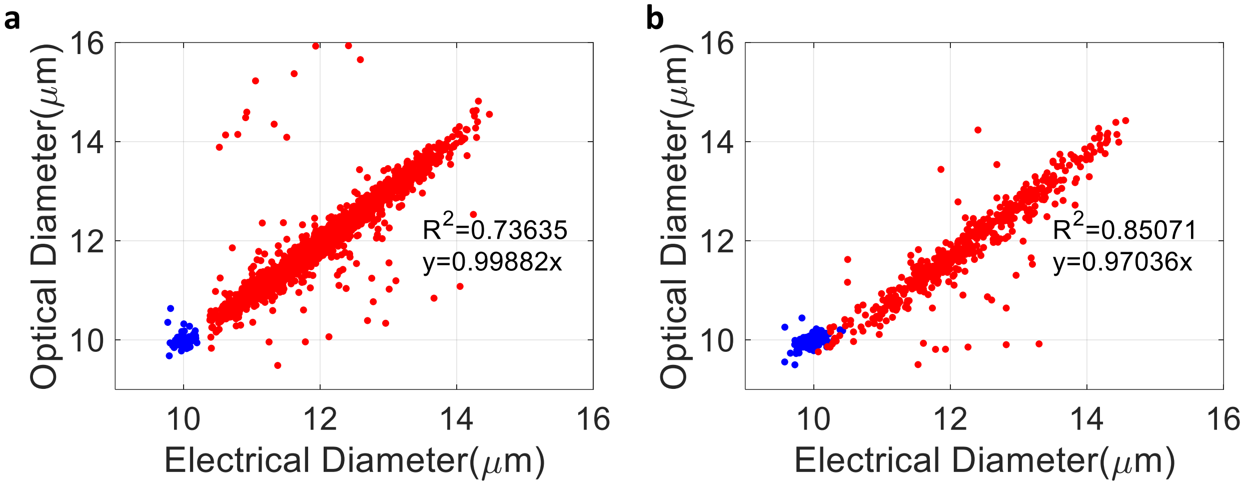

Figure S1 Correlation of optical and electrical diameter for untreated HL60 cells at (a) 10ul/min and (b) 15ul/min. Data was obtained for the same cells as the correlation plot in the text (Figure 3c) but with different flow rates.

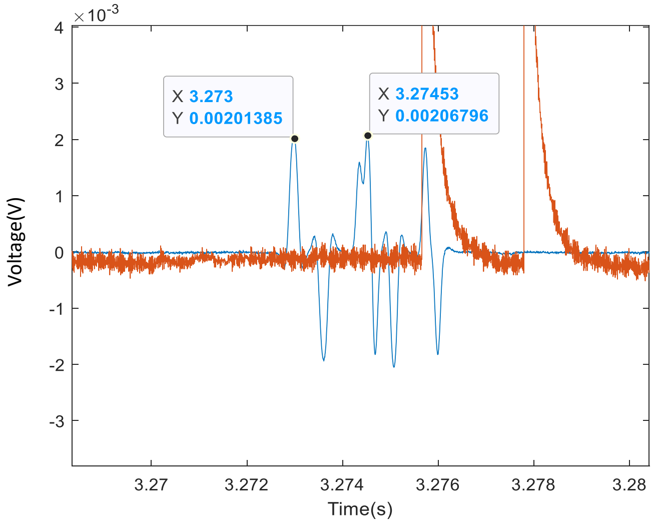

Figure S2 Example of impedance coincidence with the corresponding trigger signal. The time interval between these two particular events is 1.53 ms.
